# Evaluating the Performance of Four Risk Assessment Scores in Nonvariceal Upper Gastrointestinal Bleeding

**DOI:** 10.7759/cureus.86515

**Published:** 2025-06-22

**Authors:** Omer Kheir, Mohammed Ghamdi, Sheikha Dossary, Anwar B Alotaibi, Elrasheed M Elsabani, Hanin Fahad, Mona Alfaifi

**Affiliations:** 1 Population Health, Johns Hopkins Aramco Healthcare, Dhahran, SAU; 2 Research, Johns Hopkins Aramco Healthcare, Dhahran, SAU; 3 Hospital Medicine, Johns Hopkins Aramco Healthcare, Dhahran, SAU; 4 Epidemiology and Biostatistics, Princess Nourah Bint Abdulrahman University, Riyadh, SAU

**Keywords:** bar, lta, national early warning score (news), nonvariceal upper gastrointestinal bleeding, ptar, risk stratification, upper gastrointestinal bleeding

## Abstract

Introduction: Nonvariceal upper gastrointestinal bleeding (NVUGIB) continues to remain a life-threatening medical emergency, resulting in significant morbidity and mortality. Several scoring systems have been developed to predict outcomes, but the best risk stratification tool for emergency situations remains uncertain. This study examines four existing scoring systems: the blood urea nitrogen-to-serum albumin ratio (BAR), the international normalized ratio-to-albumin ratio (PTAR), the lactate-to-albumin ratio (LTA), and the National Early Warning Score (NEWS).

Methods: This retrospective, hospital-based study was conducted at the Johns Hopkins Aramco Healthcare (JHAH) facility in eastern Saudi Arabia from January 2020 to September 2023. Eligible participants were non-trauma UGIB patients aged 18 years or older. Cancer patients were excluded. Patient characteristics, vital signs, test results, comorbidities, disposition, and survival status at discharge were among the information collected from medical records. The four score systems were calculated and compared based on their predictive performance.

Result: NEWS demonstrated the highest overall predictive performance, particularly for hospital admission (AUC 84%) and 90-day mortality (AUC 77.2%). Both NEWS and BAR were equally effective in predicting blood transfusion (AUC 71.7%). LTA showed the highest sensitivity for mortality prediction (75%), while PTAR provided moderate predictive value across all outcomes.

Conclusion: Among the four scoring systems evaluated, NEWS consistently outperformed others in predicting key clinical outcomes in NVUGIB, making it a reliable tool for initial risk stratification. BAR is also valuable for assessing transfusion needs, while LTA and PTAR may serve complementary roles depending on the clinical context.

## Introduction

Nonvariceal upper gastrointestinal bleeding (NVUGIB) is a life-threatening medical emergency with significant morbidity and mortality [1]. Moreover, Alali and Barkun's research pointed out that "ulcers are the most common etiology of NVUGIB", accounting for more than 250,000 hospital admissions yearly in the United States, with a readmission rate of 14.6% [2]. Over the last several decades, mortality due to NVUGIB has fallen dramatically, from 4.5% in 1989 to 2.1% in 2009 [3]. Although the incidence is declining, NVUGIB remains a significant health issue, particularly in older and high-risk patients [4].

Scoring systems have emerged as crucial instruments in evaluating patients with NVUGIB. Four contemporary scoring systems have demonstrated promise in risk assessment: the blood urea nitrogen-to-serum albumin ratio (BAR) [5], international normalized ratio-to-albumin ratio (PTAR) [6], lactate-to-albumin ratio (LTA) [7], and National Early Warning Score (NEWS) [8].

The BAR has been recognized as a prognostic indicator in many critical medical conditions, such as gastrointestinal (GI) hemorrhage. A research study compared the association between BAR and prognosis in patients with GI bleeding. The results indicated that elevated BAR is correlated with high mortality and may be used as an independent risk factor for adverse prognosis in these patients [9].

Furthermore, the BAR ratio combines measurements of blood urea nitrogen (BUN) and serum albumin levels to assess a patient's nutritional and renal status, both of which can influence UGIB outcomes. It is a ratio calculated by dividing BUN levels by serum albumin levels. The BAR ratio has been shown to be associated with elevated mortality among diverse clinical conditions, including UGIB. For instance, a study proved that a BAR ratio ≥0.249 was independently associated with an increase in in-hospital mortality [10]. In another study, the authors demonstrated that increased BAR ratio at the start of treatment was strongly associated with increased risk for 30-day mortality in patients with heart failure [11].

Choi et al. found that the PTAR, reflecting both coagulation and nutritional/hepatic status, is a reliable independent predictor of ICU admission and mortality in UGIB patients, showing strong diagnostic accuracy compared to the Rockall score, Glasgow-Blatchford score (GBS), and AIMS65 score [12].

NVUGIB remains a prevalent medical emergency, resulting in significant morbidity, mortality, and healthcare resource utilization [13]. Despite breakthroughs in endoscopic procedures and medical treatment, fatality rates from UGIB have remained mainly stable in recent decades, ranging between 6% and 14% [14]. The capacity to quickly risk-stratify patients with NVUGIB upon presentation is critical for deciding the appropriate level of care and timing of endoscopic intervention and identifying high-risk patients who may benefit from intense monitoring and aggressive resuscitation [14].

Various risk assessment methods have been developed to predict outcomes in UGIB patients using clinical, biochemical, and endoscopic criteria. These methods classify patients based on their risk of unfavorable outcomes, such as rebleeding, intervention need, and mortality. Traditional systems include the pre-endoscopic Rockall score; the GBS, which includes pulse rate, systolic blood pressure, BUN, hemoglobin, and other factors; and the AIMS65 score, which includes albumin levels, an international normalized ratio (INR), altered mental status, systolic blood pressure, and age [15]. However, these methods are complex and primarily designed for UGIB, potentially restricting their universal usefulness in emergency settings.

In recent years, the LTA has gained importance as a prognostic indicator in various serious medical conditions. Lactate is a well-known indicator of tissue hypoperfusion, and increased levels have been linked to adverse outcomes in UGIB patients. Serum albumin, a negative acute-phase protein, indicates nutritional status and chronic inflammatory diseases, both of which influence bleeding outcomes. The LTA has been demonstrated to outperform other techniques (BAR and AIMS65) for predicting ICU admissions and in-hospital mortality in GI bleeding patients [7]. 

The NEWS is a risk stratification tool used in healthcare to detect early clinical deterioration. Developed by the Royal College of Physicians in 2012, it is widely adopted due to its simplicity and efficacy [8]. A modified version, including lactate levels, has been found to perform better discriminatively than the pre-endoscopic Rockall score and was comparable to the GBS and AIMS65 scores in predicting composite outcomes in UGIB patients [15]. Despite the availability of various scoring systems, no single tool has proven superior for all clinical outcomes in patients with NVUGIB. The British Society of Gastroenterology consensus care bundle recommends calculating the GBS at presentation, but other scoring systems may have value in specific contexts [14]. Direct comparisons between established scoring systems and newer biomarker ratios remain limited in NVUGIB patients.

The best scoring system for predicting outcomes in NVUGIB in emergency settings remains uncertain, despite the availability of risk stratification methods like the GBS and AIMS65. These systems can be complex, require endoscopic findings, or were developed for specific patient groups [7,15].

Few studies have directly compared recent biomarker ratios, such as the LTA and BAR, with established clinical scoring systems, such as the NEWS, in the same NVUGIB cohort [7,15].

The study aims to address the knowledge gap in NVUGIB risk assessment tools by evaluating the four different scoring systems: BAR [5], PTAR [6], LTA [7], and NEWS [8]. It aims to provide emergency physicians and gastroenterologists with evidence-based guidance on the most effective risk stratification approach for optimizing patient outcomes and resource allocation in NVUGIB. Recent studies reveal that biomarker ratios can capture both acute physiological derangement and baseline health status, improving prognostic accuracy compared to standard scores.

## Materials and methods

Study design, settings, and participants

A retrospective, hospital-based study was conducted at the Johns Hopkins Aramco Healthcare (JHAH) facility, which serves employees of Arabian American Oil Company (ARAMCO) and their dependents. Most of the participants in the research were residents of Dhahran, Al-Hasa, Ras Tanura, Abqaiq, and Udhailiyah in the eastern part of Saudi Arabia. To be eligible, participants had to be aged 18 years or older with non-traumatic upper gastrointestinal bleeding (UGIB). Patients were assessed upon admission to the emergency department, and diagnosis was confirmed based on clinical presentations including blood in the nasogastric aspirate, hematemesis, melena, or coffee ground vomit. To be eligible, participants had to be aged 18 years or older with non-traumatic UGIB. Patients were assessed upon admission to the emergency department, and diagnosis was confirmed based on clinical presentations including blood in the nasogastric aspirate, hematemesis, melena, or coffee ground vomit. At the time of their UGIB diagnosis, these individuals were considered eligible for the study. Any participant with an active or history of cancer diagnosis or a history of malignancy (as determined by biopsy results and medical records) was disqualified. This exclusion was made because cancer and its treatments may significantly influence physiological parameters and inflammatory responses, potentially interfering with the study's primary objectives.

The sample size of 227 patients was calculated using Epi Info (Centers for Disease Control and Prevention, Atlanta, Georgia, United States; https://www.cdc.gov/epiinfo/index.html), assuming a 95% confidence level and an estimated UGIB prevalence of 23% [16]. A total of 229 participants were included in this study.

Data extraction and statistical analysis

Data were extracted from medical health records at JHAH for patients diagnosed with UGIB between January 2020 and September 2023. A data dictionary was used to collect the following variables: patient demographics (age, sex), vital signs, laboratory findings (hemoglobin, albumin level, BUN, prothrombin time (PT), INR, and lactate level), comorbidities, disposition (discharge, admission to ward, or ICU), and survival status at hospital discharge. The data were validated by randomly selecting 10% of records and cross-checking them against the original medical records, with discrepancies resolved by a second reviewer.

Key metrics calculated included the BAR [5], PTAR [6], LTA [7], and NEWS [8]. BAR, PTAR, and LTA were selected as novel markers of UGIB severity based on prior studies [7,8], while NEWS was used to assess clinical deterioration [8]. These metrics were calculated using standardized formulas implemented in Microsoft Excel (Microsoft Corporation, Redmond, Washington, United States). Statistical analyses were performed to evaluate associations between these markers and patients' outcomes, with methods detailed in the subsequent section.

Demographic and clinical characteristics (n=229) were summarized using means with standard deviations for continuous variables (e.g., age, vital signs) and frequencies with percentages for categorical variables (e.g., symptoms, disposition). Receiver operating characteristic (ROC) curve analysis evaluated the predictive performance of BAR, PTAR, LTA, and NEWS for blood transfusion, inpatient admission, and 90-day mortality. Area under the curve (AUC) with 95% confidence intervals (CIs) assessed discriminatory ability, with statistical significance tested using the DeLong test (p<0.05 threshold). Optimal cut-off points were determined via the Youden index, and sensitivity, specificity, positive predictive value (PPV), and negative predictive value (NPV) were calculated. Analyses were performed using R (Version 4.3.1, R Foundation for Statistical Computing, Vienna, Austria) with pROC and stats packages; scoring system metrics were calculated in Microsoft Excel using standardized formulas.

## Results

The demographic and clinical characteristics of 229 patients are included in this study. Notably, this patient population is the same as that described in our previous publication, which focused on risk scoring systems in NVUGIB [17]. The mean age of the population was 68.29±16.05 years, with a slight male predominance (n=122; 53.28%). Abdominal pain was the most common presenting symptom (n=159; 69.43%), followed by rectal bleeding (n=28; 12.23%) and vomiting blood (n=26; 11.35%). Most patients were alert at presentation (n=199; 86.9%) with stable vital signs; the mean systolic blood pressure was 129.71±33.26 mmHg, and the mean oxygen saturation was 98.88%±0.46. Blood transfusion was required in 57 patients (24.89%), while inpatient admission occurred in 179 (78.51%). The observed overall mortality rate was 22.71% (n=52), and the 90-day mortality rate was 8.73% (n=20).

**Figure 1:**
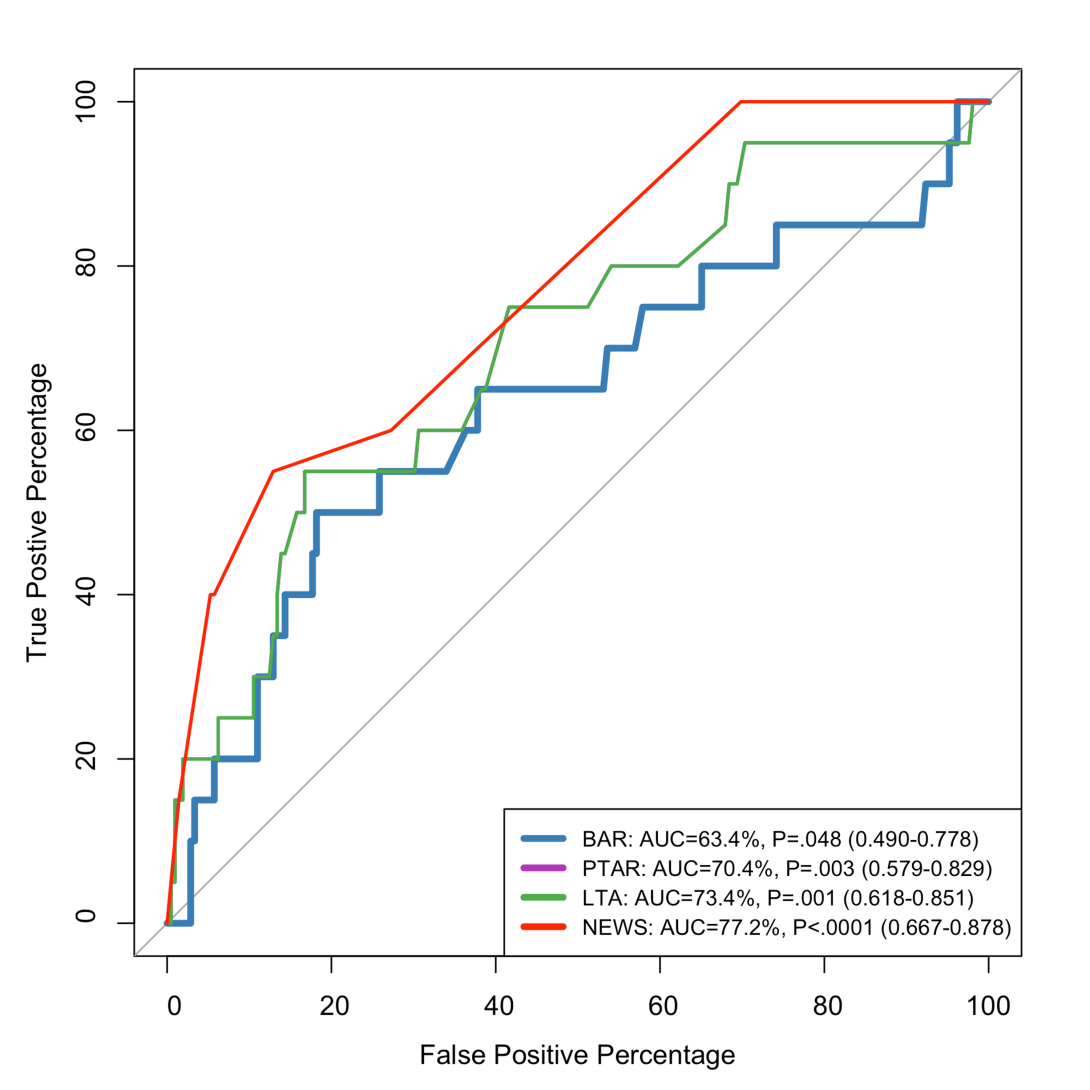
ROC curve for predicting the 90-day mortality ROC: receiver operating characteristic; BAR: blood urea nitrogen-to-serum albumin ratio; PTAR: international normalized ratio-to-albumin ratio; LTA: lactate-to-albumin ratio; NEWS: National Early Warning Score; AUC: area under the curve

Figure 1 illustrates the ROC curves for predicting the 90-day mortality. NEWS demonstrates the best performance with an AUC of 77.2% and high statistical significance (p<0.0001; 95% CI: 0.667-0.878). Its curve shows a pronounced shift toward the upper-left corner, indicating excellent discriminatory ability with high true positive rates even at low false positive rates. LTA follows as the second most effective predictor with an AUC of 73.4% (p=0.001; 95% CI: 0.618-0.851), showing good but less discriminatory ability compared to NEWS. PTAR shows moderate effectiveness with an AUC of 70.4% (p=0.003; 95% CI: 0.579-0.829), ranking third in reliability but still providing statistically significant predictive value (Figure 1). BAR demonstrates the weakest performance with an AUC of 63.4% (p=0.048; 95% CI: 0.490-0.778). While still statistically significant (though marginally at p=0.048), its lower AUC indicates less reliable discriminatory power compared to the other systems (Figure 1).

**Figure 2:**
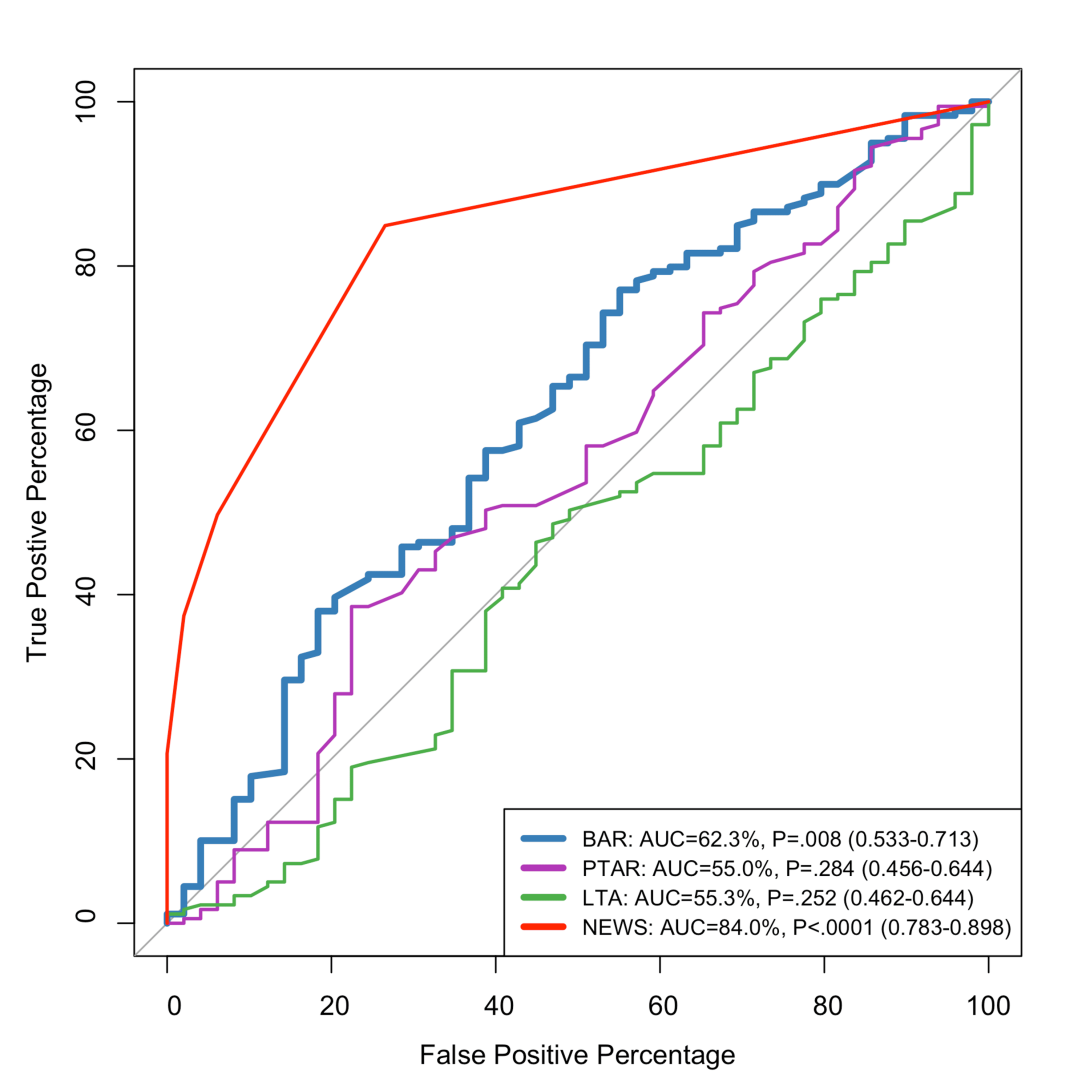
ROC curve for predicting inpatient admission ROC: receiver operating characteristic; BAR: blood urea nitrogen-to-serum albumin ratio; PTAR: international normalized ratio-to-albumin ratio; LTA: lactate-to-albumin ratio; NEWS: National Early Warning Score; AUC: area under the curve

The ROC curve examination of scoring systems for predicting hospital admission in GI bleeding patients shows considerable performance differences among NEWS, LTA, BAR, and PTAR. NEWS is highly predictive, with an AUC of 84% (p<0.0001; 95% CI: 0.783-0.898). The BAR scoring system is moderately effective, with an AUC of 62.3% (p=0.008; 95% CI: 0.533-0.713). While statistically significant, the curve is substantially lower for the majority of the graph. PTAR and LTA both perform poorly. LTA shows comparable limitations with an AUC of 55.3% (p=0.252; 95% CI: 0.462-0.644), while PTAR has an AUC of 55% (p=0.284; 95% CI: 0.456-0.644) (Figure 2). 

**Figure 3:**
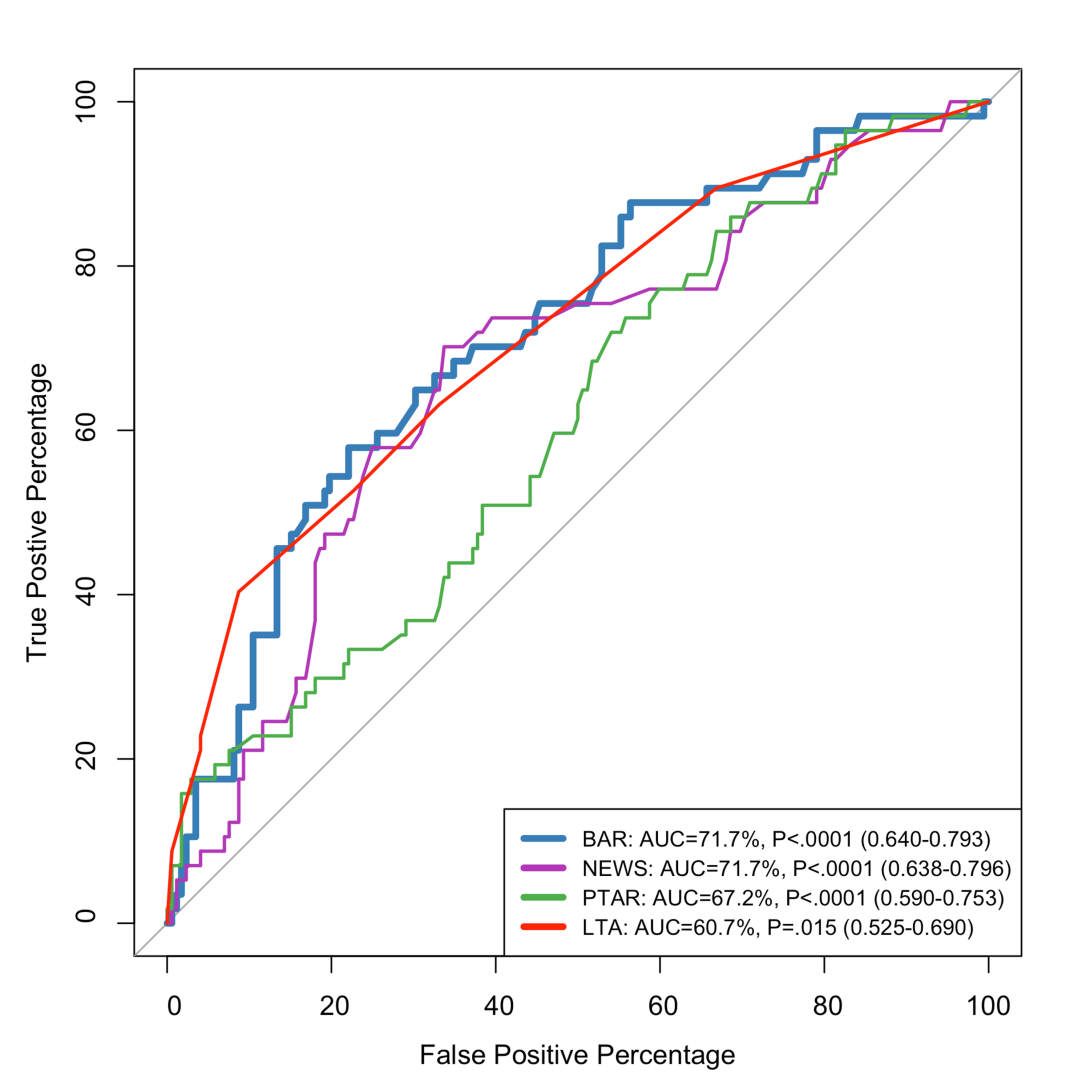
ROC curve for predicting blood transfusion ROC: receiver operating characteristic; BAR: blood urea nitrogen-to-serum albumin ratio; PTAR: international normalized ratio-to-albumin ratio; LTA: lactate-to-albumin ratio; NEWS: National Early Warning Score; AUC: area under the curve

Figure 3 displays the ROC curves for predicting the need for blood transfusion. Both the BAR and the NEWS demonstrated the highest diagnostic accuracy with identical AUCs of 71.7% (p<0.0001), indicating good discriminatory power. The PTAR followed with an AUC of 67.2% (p<0.0001), showing moderate performance. The LTA had the lowest AUC at 60.7% (p=0.015), suggesting limited predictive utility. These findings support the use of BAR and NEWS as effective tools for early risk assessment in NVUGIB, with PTAR showing potential as a novel marker, while LTA may be less reliable in this context (Figure 3). 

Table 1 summarizes the predictive performance of four scoring systems in NVUGIB. For blood transfusion, PTAR showed high sensitivity (70.18%) and relatively high specificity (66.28%), indicating a good balance in identifying patients likely to require transfusion. LTA also showed high sensitivity (73.68%) but lower specificity (44.19%). In predicting inpatient admission, NEWS had the highest sensitivity (84.92%) and specificity (73.47%), making it the most effective tool for identifying patients needing hospitalization. Regarding 90-day mortality, LTA achieved the highest sensitivity (75%) and strong specificity (63.64%), with the highest negative predictive value (96.38%), indicating its potential as a reliable prognostic marker. PTAR showed moderate sensitivity (55%) and good specificity (83.25%), suggesting it may be useful for identifying patients at lower risk of death. Overall, the results suggest that while BAR and PTAR are useful for predicting transfusion need, NEWS excels in anticipating admission, and LTA may be the most reliable for identifying patients at risk of mortality.

**Table 1:** Predictive performance of risk scores for upper gastrointestinal bleeding outcomes BAR: blood urea nitrogen-to-serum albumin ratio; PTAR: international normalized ratio-to-albumin ratio; LTA: lactate-to-albumin ratio; NEWS: National Early Warning Score; PPV: positive predictive value; NPV: negative predictive value

Outcome	Predictor	Cut-off point	Sensitivity	Specificity	PPV	NPV
Blood transfusion	BAR	12.90	57.89%	78.91%	46.48%	84.81%
	PTAR	0.39	70.18%	66.28%	40.82%	87.02%
	LTA	0.43	73.68%	44.19%	30.43%	83.52%
	NEWS	4.00	40.35%	77.25%	60.53%	82.2%
Inpatient admission	BAR	4.52	77.1%	44.9%	83.64%	34.92%
	PTAR	0.41	38.55%	77.55%	86.25%	25.68%
	LTA	0.48	48.6%	53.06%	79.09%	22.03%
	NEWS	1.00	84.92%	73.47%	92.12%	57.14%
90-day mortality	BAR	17.04	50%	81.82%	20.83%	94.48%
	PTAR	0.47	55%	83.25%	23.91%	95.08%
	LTA	0.58	75%	63.64%	16.48%	96.38%
	NEWS	4.00	55%	87.08%	28.95%	95.29%

## Discussion

We compared and evaluated the predictive validity of four scores (BAR [5], PTAR [6], LTA [7], and NEWS [8]) for serious clinical outcomes among patients presenting initially with NVUGIB. Our findings confirmed that BAR and NEWS were the most predictive of blood transfusion, with similar AUCs of 71.7% (p<0.0001). This aligns with prior studies indicating BAR's prognostic value in GI bleeding, as higher BAR reflects impaired renal function and malnutrition, both associated with worse outcomes [9]. Similarly, NEWS has been recognized for its ability to detect early physiological deterioration through changes in vital signs. These changes, which reflect the body's response to significant blood loss, explain why NEWS is closely associated with the need for blood transfusions. Our results further support its utility in the setting of UGIB, consistent with its performance in other acute medical conditions [8].

In predicting inpatient admission, NEWS demonstrated the highest sensitivity (84.92%) and a strong specificity (73.47%) compared to other scoring systems. For example, while the BAR score showed relatively high sensitivity (77.1%), it had poor specificity (44.9%), potentially leading to overestimation and unnecessary admissions. On the other hand, PTAR had better specificity (77.55%) but very low sensitivity (38.55%), indicating a high rate of missed admissions. LTA performed poorly in both metrics. These limitations explain why other scores were less reliable for accurately predicting inpatient needs. This aligns with Kim et al., who reported that NEWS provides excellent discrimination in identifying hospitalization needs among UGIB patients, performing as well as or better than traditional risk scores such as GBS and AIMS65 [15].

In terms of 90-day mortality rate, LTA was most sensitive (75%) with a high NPV (96.38%), suggesting its use as a good prognostic indicator in the long term. This is corroborated by current research, where the LTA has been demonstrated to be a significant predictor of adverse outcomes in critically ill patients, particularly in those with UGIB, as it is a marker of tissue hypoperfusion and systemic inflammation [7]. In spite of PTAR demonstrating a moderate ability to predict mortality (AUC 67.2%), it showed a strong specificity (83.25%) in keeping with previous studies indicating the combined impact of both coagulopathy and hypoalbuminemia in the severity and outcome of UGIB [12].

In general, our results suggest that while BAR and NEWS are good in early risk stratification for hospitalization and transfusion of blood, LTA can be a good prognostic indicator of mortality. PTAR has moderate performance over a range of outcomes and may be a good pragmatic marker, particularly in resource-limited areas where rapid laboratory tests are crucial. These findings contribute to the growing evidence supporting the addition of biomarker-derived ratios to clinical scoring systems for optimizing the initial management of NVUGIB [5,7,12].

NEWS' exceptional performance for hospital admission prediction (AUC 84%), exceeding BAR, PTAR, and LTA, is consistent with Williams' observations about its broad clinical utility since NHS adoption [8]. The moderate performance of BAR (AUC 62.3%) for admission prediction in our investigation is consistent with Aktas et al.'s comparative examination of scoring systems for NVUGIB, which discovered varying performance across different scoring tools when applied to admission decisions [1].

In the context of NVUGIB, the comparable performance of NEWS and BAR for blood transfusion prediction (both AUC 71.7%) is an intriguing observation. The alignment of a general physiological scoring system (NEWS) and a bleeding-specific instrument (BAR) reveals that both overall physiological derangement and bleeding-specific markers play an equal role in transfusion decisions in NVUGIB patients. This study confirms Siau et al.'s British Society of Gastroenterology (BSG)-led multisociety consensus care bundle recommendations, which emphasize taking into account both general patient state and bleeding-specific factors while managing acute UGIB [14].

Our analysis revealed PTAR's moderate performance for transfusion prediction (AUC 67.2%) but poor performance for admission prediction (AUC 55%). The varying efficacy of PTAR is consistent with Simoens and Rutgeerts' early finding that risk stratification methods for NVUGIB must be carefully studied for their intended results [13]. Our findings show that PTAR may be limited in predicting admission needs for NVUGIB patients. This performance variation is consistent with recommendations from the BSG-led multisociety agreement, which recognizes that different scoring systems may serve different purposes in the treatment pathway for acute UGIB [14]. Furthermore, Kim et al.'s comparative analysis of various scoring systems revealed that traditional bleeding-specific scores and physiological parameters may perform differently depending on the specific outcome being predicted [11], which supports our observation of PTAR's outcome-dependent performance.

LTA's consistently poor performance across all outcomes (AUC 55.3% for admission; 60.7% for transfusion) contradicts Bae et al.'s recent findings that the LTA may serve as a predictive tool in emergency department patients with GI bleeding [7]. This disparity demonstrates that good predictive performance for one outcome does not necessarily translate into efficacy in predicting other clinical endpoints. Although LTA has shown promise in other bleeding contexts, the discrepancies between our findings and those of other studies are likely due to differences in study populations and methodological approaches. This suggests that LTA's efficacy may be limited in the NVUGIB population.

The Saudi population was the focus of this study, which also offered helpful insights into how various scoring systems function in a group with a variety of clinical and healthcare characteristics. This study did, however, have a number of limitations. The study should be addressed because it was a retrospective observational single study. One significant issue that has to be acknowledged is the uncertainty around the generalizability to other circumstances, such as various hospitals, geographical areas, or countries. This single-center study has inherent limitations in terms of broader application due to potential differences in clinical practices, patient demographic profiles, and healthcare system structure that occur across various settings. Major limitations of our study include incomplete data on endoscopic interventions and rebleeding episodes. The absence of comprehensive intervention and outcome data could significantly affect the validity of our predictions and potentially introduce bias in the observed associations, as treatment patterns and their effectiveness may have influenced the clinical endpoints we measured. In order to optimize clinical decision-making, future research could look into combining these scores into a single prediction model and utilizing their individual advantages. Furthermore, this combined approach could provide doctors with an excellent tool for managing UGIB in a range of patient categories. 

## Conclusions

Our findings demonstrate that four commonly used scoring systems, namely, NEWS, LTA, PTAR, and BAR, have differing levels of effectiveness when predicting significant clinical implications for individuals experiencing NVUGIB. The NEWS had the best predictive accuracy when it came to hospital admission prediction. The NEWS and the BAR both performed well in predicting transfusion needs. The NEWS demonstrated higher predictive capacity in predicting 90-day mortality. These findings indicate that the scoring system should be adapted to the individual clinical outcome of interest, with the NEWS providing the best consistent performance across multiple endpoints. Based on these findings, healthcare facilities should consider using the NEWS as their standard monitoring system while acknowledging that customization may be beneficial for specific patient populations or clinical scenarios in which specific outcomes are of primary concern.
